# Adult Caregivers in the United States: Characteristics and Differences in Well-being, by Caregiver Age and Caregiving Status

**DOI:** 10.5888/pcd10.130090

**Published:** 2013-08-15

**Authors:** Lynda A. Anderson, Valerie J. Edwards, William S. Pearson, Ronda C. Talley, Lisa C. McGuire, Elena M. Andresen

**Affiliations:** Author Affiliations: Valerie J. Edwards, William S. Pearson, Lisa C. McGuire, Centers for Disease Control and Prevention, Atlanta, Georgia; Ronda C. Talley, Western Kentucky University, Bowling Green, Kentucky; Elena M. Andresen, Oregon Health and Science University, Portland, Oregon.

## Abstract

We examined the characteristics of adults providing regular care or assistance to friends or family members who have health problems, long-term illnesses, or disabilities (ie, caregivers). We used data from the 2009 Behavioral Risk Factor Surveillance System (BRFSS) to examine caregiver characteristics, by age and caregiving status, and compare these characteristics with those of noncaregivers. Approximately 24.7% (95% confidence interval, 24.4%–25.0%) of respondents were caregivers. Compared with younger caregivers, older caregivers reported more fair or poor health and physical distress but more satisfaction with life and lower mental distress. Understanding the characteristics of caregivers can help enhance strategies that support their role in providing long-term care.

## Objective

In 2007, Talley and Crews framed caregiving as a public health issue and argued for its inclusion in state-level surveillance to obtain data about adult caregivers (1). Subsequently, a caregiving question was added to the 2009 Behavioral Risk Factor Surveillance System (BRFSS) survey. Respondents were asked whether they provided regular care or assistance to friends or family members who have health problems, long-term illnesses, or disabilities during the past month. The objective of this study was to examine the demographic characteristics of caregivers, compare caregivers with noncaregivers, and compare younger (aged 18–64) and older (aged ≥65) caregivers on measures of well-being.

## Methods

The BRFSS is an annual, state-based telephone survey (2). Data are from a representative sample of noninstitutionalized people aged 18 years or older from 50 states, the District of Columbia, and 2 US territories. Responses are weighted to the estimated population in states or territories. The median cooperation rate for the 2009 survey was 75.0%, and the median Council of American Survey Research Organizations response rate was 52.5%. A complete description of 2009 BRFSS procedures and definitions of response rates are available at www.cdc.gov/brfss/technical_infodata/surveydata/2009.htm.

Respondents were classified as caregivers if they responded yes to the following question added to the 2009 BRFSS core: “People may provide regular care or assistance to a friend or family member who has a health problem, long-term illness, or disability. During the past month, did you provide any such care or assistance to a friend or family member?” Estimates were calculated for prevalence of caregiving by demographic measures (age, sex, race/ethnicity, education, marital status, and employment status) using pairwise deletion. Ninety-five percent confidence intervals (CIs) were provided for each estimate, and SPSS software version 20 (IBM Corp, Armonk, New York) was used to account for the BRFSS’s complex sample design.

Respondents were classified as having frequent physical distress if they reported they had experienced 14 or more days in the past month when their physical health interfered with their daily activities. Respondents were classified as having frequent mental distress if they reported 14 or more days when their mental health interfered with daily activities. We compared caregivers and noncaregivers on self-reported, health-related quality of life, degree of life satisfaction, and availability of emotional support. Finally, we compared older caregivers with younger caregivers on these same health and well-being measures. We used the Rao-Scott adjusted χ^2^ statistic to determine the independence of all comparisons.

## Results

Overall, 24.7% (95% CI, 24.4%–25.0%) of respondents to the 2009 BRFSS were classified as caregivers. Caregivers were significantly more likely to be 50 to 64 years of age than from other age groups, to be female than male, and to be non-Hispanic black than from other racial/ethnic groups (Table 1). Caregivers were significantly more likely to have some college education than to have a high school education or less or to be a college graduate; they were also significantly more likely to be married or part of an unmarried couple than to be divorced, separated, widowed, or never married (Table 1).

**Table 1:** Weighted Estimates of Caregivers,^a^ by Selected Demographics, in the United States, District of Columbia, and 2 US Territories, Behavioral Risk Factor Surveillance System (BRFSS), 2009

Characteristic	% Weighted^b^ Estimate (95% Confidence Interval)	*P* Value^c^
**Age, y**		
18–34	20.6 (19.9–21.2)	<.001
35–49	25.8 (25.2–26.3)	
50–64	30.5 (30.1–31.0)	
≥65	21.6 (21.2–22.0)	
**Sex**		
Male	22.0 (21.6–22.5)	<.001
Female	27.2 (26.9–27.6)	
**Race/ethnicity**		
White, non-Hispanic	24.8 (24.6–25.1)	<.001
Black, non-Hispanic	28.6 (27.5–29.7)	
Other race or multiracial, non-Hispanic	24.0 (22.6–25.4)	
Hispanic	21.4 (20.5–22.5)	
**Education**		
Less than high school	20.4 (19.4–21.4)	<.001
High school graduate	24.5 (24.0–25.1)	
Some college	26.9 (26.3–27.4)	
College graduate	24.5 (24.0–24.9)	
**Marital status**		
Married/part of unmarried couple	25.3 (24.9–25.6)	<.001
Divorced, separated, or widowed	24.3 (23.8–24.9)	
Never married	24.7 (24.4–25.0)	
**Employment status**		
Employed for wages or self-employed	24.6 (24.2–25.0)	.56
Not employed	24.8 (24.3–25.2)	
Unable to work	25.3 (24.0–26.6)	

a Respondents were classified as caregivers if they answered yes to the following question: “People may provide regular care or assistance to a friend or family member who has a health problem, long-term illness, or disability. During the past month, did you provide any such care or assistance to a friend or family member?”

b Information about BRFSS weighting procedures can be found at http://www.cdc.gov/brfss/about/brfss_faq.htm#15.

c *P* value calculated using the Rao-Scott adjusted χ^2^ statistic.

Comparing caregivers with noncaregivers, significantly more caregivers reported fair or poor self-rated health (16.9% [95% CI, 16.4%–17.3%] vs 15.8% [95% CI, 15.5%–16.0%]), more frequent physical distress (12.0% [95% CI, 11.6%–12.4%] vs 10.5% [95% CI, 10.3%–10.7%]), more frequent mental distress (14.3% [95% CI, 13.9%–14.8%] vs 9.4% [95% CI, 9.2%–9.6%]), and being dissatisfied or very dissatisfied with life (7.0% [95% CI, 6.7%–7.4%] vs 5.5% [95% CI, 5.3%–5.7%]) than noncaregivers. Caregivers and noncaregivers did not differ, however, on the reported availability of social support (8.5% [95% CI, 8.1%–8.9%] vs 8.5% [95% CI, 8.2%–8.7%]).

Compared with younger caregivers, those aged 65 or older reported a higher prevalence of fair or poor self-rated health and more frequent physical distress compared with caregivers aged 18 to 64 years (Table 2). Caregiver age was not related to reports of availability of emotional support. Caregivers aged 65 or older reported lower prevalence of frequent mental distress and being dissatisfied or very dissatisfied with their life compared with caregivers aged 18 to 64 years (Table 2).

**Table 2:** Weighted Estimates of Measures of Self-Reported Health and Well-being of Caregivers, by Age, Behavioral Risk Factor Surveillance System (BRFSS), 2009

Measure	Prevalence Aged 18–64 (95% CI)	Prevalence Aged ≥65 (95% CI)	*P* Value^a^
Fair or poor self-rated health	15.7 (15.1–16.2)	23.8 (22.9–24.7)	<.001
Frequent mental distress	15.3 (14.8–15.9)	8.6 (8.0–9.2)	<.001
Frequent physical distress	11.5 (11.0–11.9)	15.0 (14.3–15.8)	<.001
Rarely/never get emotional support	8.5 (8.0–8.9)	8.7 (8.1–9.3)	.56
Dissatisfied/very dissatisfied with life	7.6 (7.2–8.0)	3.8 (3.4–4.2)	<.001

Abbreviation: CI, confidence interval.

a *P* value calculated using the Rao-Scott adjusted χ^2^ statistic.

## Discussion

Approximately one-quarter of respondents were classified as caregivers. Estimates of the prevalence of caregivers vary across studies because of differences in definitions, sampling, and the age of person receiving care (3). For example, the 2000 BRFSS found a prevalence of 16.4% (standard error, 0.2%), but care was restricted to “a person aged 60 years or older” (4). Our higher prevalence may be due to elimination of the age restriction or other changes in the population and requires replication. We found that people classified as caregivers reported decrements in health-related quality of life compared with noncaregivers. We found that younger caregivers reported more mental distress compared with older caregivers. This finding is consistent with those of previous studies (3,5).

The findings are subject to several limitations. Many factors may affect participation in telephone surveys and may influence our estimates of caregivers, such as their ability to participate in surveys, exclusion of institutionalized populations, and potential cultural differences about the meaning of providing care (6). We lacked data on the care recipient and the amount of time dedicated to care and type of caregiving activities. More detailed information about caregiving provision and activities is available from the BRFSS module being used in select states (5). Finally, data were collected in all 50 states, the District of Columbia, and 2 US territories and weighted to state-based population estimates.

Caregiving has gained national attention because family members have been and will continue to be the primary providers of care to those with illnesses and disabilities (7–9). Caregiving will likely increase as the US population ages (10). As a result, understanding the extent, characteristics, and effects of caregiving on health and well-being can enhance efforts to improve the quality of life of caregivers and inform national strategies such as evidence-based programs for caregivers (11) and care transitions (12).
